# Distinct distribution and prognostic significance of molecular subtypes of breast cancer in Chinese women: a population-based cohort study

**DOI:** 10.1186/1471-2407-11-292

**Published:** 2011-07-12

**Authors:** Yinghao Su, Ying Zheng, Wei Zheng, Kai Gu, Zhi Chen, Guoliang Li, Qiuyin Cai, Wei Lu, Xiao Ou Shu

**Affiliations:** 1 Department of Medicine, Vanderbilt Epidemiology Center and Vanderbilt-Ingram Cancer Center, Vanderbilt University Medical Center, 2525 West End Ave, Suite 600, Nashville, TN 37203-1738, USA; 2 Shanghai Institute of Preventive Medicine, 1380 Zhong Shan Road (W), Shanghai 200336, China

## Abstract

**Background:**

Molecular classification of breast cancer is an important prognostic factor. The distribution of molecular subtypes of breast cancer and their prognostic value has not been well documented in Asians.

**Methods:**

A total of 2,791 breast cancer patients recruited for a population-based cohort study were evaluated for molecular subtypes of breast cancer by immunohistochemical assays. Data on clinicopathological characteristics were confirmed by centralized pathology review. The average follow-up of the patients was 53.4 months. Overall and disease-free survival by molecular subtypes of breast cancer were evaluated.

**Results:**

The prevalence of the luminal A, luminal B, human epidermal growth factor receptor 2 (HER2), and triple-negative subtypes were 48.6%, 16.7%, 13.7%, and 12.9%, respectively. The luminal A subtype was more likely to be diagnosed in older women (P = 0.03) and had a stronger correlation with favorable clinicopathological factors (smaller tumor size, lower histologic grade, and earlier TNM stage) than the triple-negative or HER2 subtypes. Women with triple-negative breast cancer had a higher frequency of family history of breast cancer than women with other subtypes (P = 0.048). The 5-year overall/disease-free survival percentages for the luminal A, luminal B, HER2, and triple-negative subtypes were 92.9%/88.6%, 88.6%/85.1%, 83.2%/79.1%, and 80.7%/76.0%, respectively. A similar pattern was observed in multivariate analyses. Immunotherapy was associated with improved overall and disease-free survival for luminal A breast cancer, but reduced disease-free survival (HR = 2.21, 95% CI, 1.09-4.48) for the HER2 subtype of breast cancer.

**Conclusions:**

The triple-negative and HER2 subtypes were associated with poorer outcomes compared with the luminal A subtype among these Chinese women. The HER2 subtype was more prevalent in this Chinese population compared with Western populations, suggesting the importance of standardized HER2 detection and anti-HER2 therapy to potentially benefit a high proportion of breast cancer patients in China.

## Background

Breast cancer is highly heterogeneous with regard to morphological spectrum, clinical presentation, and response to cancer therapy [1]. Based on gene-expression profiling using cDNA microarray technology, a molecular taxonomy has been proposed to classify breast cancer into luminal A, luminal B, basal-like, and HER2 subtypes, which have distinct differences in prognosis and responses to cancer therapies [2,3]. Using conventional immunohistochemistry (IHC) detection of estrogen receptor-alpha (ERα), progesterone receptor (PR), and human epidermal growth factor receptor 2 (HER2) status, molecular subtypes of breast cancer can be classified as: luminal A (ERα+ and/or PR+, HER2-), luminal B (ERα+ and/or PR+, HER2+), triple-negative (ERα-, PR- and HER2-), and HER2 (HER2+, ERα-, and PR-) [4]. It has been suggested that the triple-negative and HER2 subtypes defined by IHC have poorer survival outcomes and respond differently to adjuvant chemotherapy compared with the luminal A subtype [4,5]. Most previous studies were conducted in Western populations, while few population-based studies have been conducted in Asians.

Racial differences in molecular subtypes have been reported. For example, the triple-negative subtype appears to be more common in African-American populations, especially among younger African-American women, compared with European-ancestry populations [4,6-8]. One study has suggested that the HER2 subtype is more common in Asian populations and that the distribution of breast cancer subtypes among Asian women may vary by ethnicity (i.e., Chinese, Japanese, etc.) [9]. A few studies have evaluated the molecular subtypes of breast cancer in Chinese women [10-14]. However, most of those studies have had a relatively small sample size and applied different criteria to define positivity. For example, HER2 has been defined as positive with a DAKO score of 3+ (>10% cells show strong complete membrane staining) [10-12,14] or ≥2+ (>10% cells show weak to moderate complete membrane staining) [13]. The widely used criteria for HER2 positivity modified by the American Society of Clinical Oncology/College of American Pathologists guidelines [15] were not used in those publications. The prevalence and clinicopathological significance of breast cancer subtypes in the Chinese population merits verification. The present study used data from a large-scale, population-based cohort study of breast cancer patients in Shanghai, China [16]. The distribution of molecular subtypes of breast cancer and their correlation with breast cancer outcomes were evaluated.

## Methods

### Participants

Study participants were women aged 20 to 75 years who were diagnosed with a primary breast cancer and enrolled in the Shanghai Breast Cancer Survival Study (SBCSS), a longitudinal, population-based cohort study in Shanghai, China [16]. Through the population-based Shanghai Cancer Registry, 6,299 women were identified approximately 6.5 months after a cancer diagnosis, and 5,042 were enrolled in the study (participation rate: 80.0%) between March 2002 and April 2006. The SBCSS was approved by the institutional review boards of all institutions involved in the study, and written informed consent was obtained from all participants.

### Data collection

Trained interviewers, all retired health professionals (e.g., nurses and physicians), conducted in-person interviews using a standard baseline survey questionnaire to collect information on demographic characteristics, reproductive history, disease history, medication use, selected lifestyle factors, diet, use of complementary and alternative medicine, and quality of life. Clinical information collected included cancer stage, tumor ERα and PR status, and primary treatments. Inpatient medical charts were reviewed to verify clinical information. Anthropometric measurements, including height, weight, and circumferences of the waist and hips, were taken according to a standard protocol by trained interviewers at the baseline interview. The cohort is being followed up by in-person interviews that take place at 18 months, 36 months, and 60 months after cancer diagnosis, supplemented by record linkage to the Shanghai Vital Statistics Registry.

### Tissue slide preparation

Pathology slides for 2,791 cases were available for this study. The slides were collected from the diagnosis hospitals according a standard protocol. Briefly, the formalin-fixed, paraffin-embedded blocks were cut in 5 μm thick sections. The sectioned tissue slides were covered with a thin layer of paraffin, and stored in vacuum chambers (Terra Universal, Inc., Anaheim, CA) placed in a 4°C cold room to properly preserve the antigenicity of the sectioned tissues. This slide storage method has been established and verified in our centralized laboratory [17]. The diagnoses and clinicopathologic data were confirmed by a combination of medical chart review and centralized review of pathology slides. The histological types of breast cancer were confirmed according to the criteria of the World Health Organization classification [18] by the study pathologist (Su). The histologic grade of all cancer slides was determined using the Nottingham histologic grading system [19].

### Immunohistochemistry

**Figure 1:**
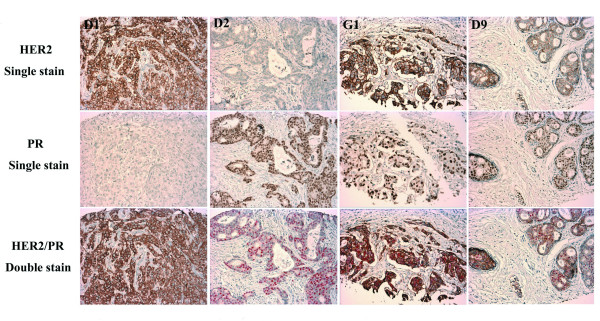
**Double immunohistochemical staining for PR/HER2**. To validate the lab staining method, commercial breast cancer tissue microarray (TMA) slides BR701 (US Biomax Inc.) were used. D1, TMA core with HER2+ and PR- staining. D2, HER2- and PR+ staining. G1, HER2+ and PR+ staining. D9, PR+ and HER2 weak-positive (borderline) staining. PR/HER2 double stains were comparable to standard single stains for HER2 and PR, although the PR signal in the double staining was somewhat weaker (original magnification: ×200).

**Figure 2:**
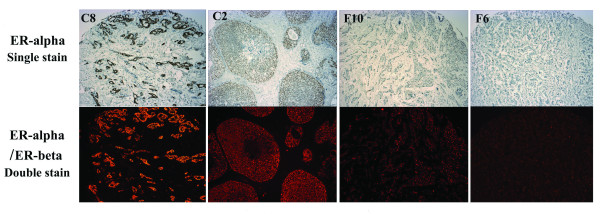
**Double immunofluorescence staining for ERα/ERβ**. The same commercial breast cancer TMA slides were used to validate ERα staining. TMA cores C2 and C8, strong ERα nuclear staining. F10, weak ERα nuclear staining. F6, negative ERα staining. ERα fluorescent positive signals were comparable to standard single staining for ERα (original magnification: ×100).

**Figure 3:**
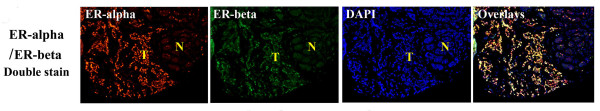
**Double immunofluorescence staining for ERα/ERβ using lab-constructed TMA control slides**. For immunostaining quality control, lab-constructed slides were stained with each batch. One TMA core of breast tissue exhibited strong ERα and ERβ nuclear staining in tumor cells (T), other than normal epithelium (N). Most tumor cells exhibited co-expression of ERα and ERβ, as revealed in the overlapping image (original magnification: ×200).

HER2 staining was conducted for all 2,791 participants included in this study in our centralized laboratory, with rabbit polyclonal antibody recognizing the HER2 cytoplasmic domain (DAKO, Cat# A0485, 1:100), following the protocol of the DAKO Envision™ kit (DAKO, Cat# K4011). This staining protocol has been validated by comparing it with the HercepTest™ kit (DAKO, Cat# K5204) using commercial tissue microarray (TMA) slides, which included tissue slides from 70 breast cancer cases (BR701, US Biomax Inc.). A 100% concordance rate between the two methods was obtained. For patients whose ERα (243 cases) and PR (222 cases) status could not be obtained from medical charts, double immunohistochemical staining for PR/HER2 and ERα/estrogen receptor beta (ERβ) was conducted. PR/HER2 double staining was performed using the EnVision™ G|2 Doublestain System (Additional file 1). ERα/ERβ double immunofluorescent staining was performed using a sequential double labeling protocol proposed by Vector Laboratories (Additional file 2). The staining protocols were carefully validated by comparison with standard staining, using the above BR701 commercial TMA breast cancer slides (Figures 1 and 2). We constructed a TMA block as a quality control, which included one breast cancer tissue sample with positive expression of ERα, ERβ, and PR; one breast cancer tissue sample with positive HER2 expression; and normal ovary, prostate, and liver tissue samples (Figure 3). The control TMA slides were stained in parallel with each batch of study samples using an Autostainer Universal Staining System (DAKO, Model LV-1).

Membrane staining intensity and the pattern of HER2 staining were evaluated using the 0 to 3+ scale [15]. Scores of 0 and 1+ (weak immunostaining in less than 30% of tumor cells) were defined as negative, 2+ (complete membranous staining, in at least 10% but less than 30% of tumor cells) as equivocal, and 3+ (uniform intense membranous staining in at least 30% of tumor cells) as positive. For ERα staining, a clinically validated threshold [20,21] for the prediction of response to hormonal therapy (>10% cutoff for whole slides) was used in this study. PR expression was considered to be positive, if the nuclei of more than 1% of cells were stained positive in a single slide. The stained slides were evaluated independently by two investigators (Su and Li), and all slides with inconsistent readings were re-evaluated by the two investigators jointly and the final status assigned.

### Statistics

Differences in sociodemographic and clinicopathologic characteristics across different breast cancer subtypes were evaluated using the one-way analysis of variance test for continuous variables (such as age), and the chi-square test for categorical variables. Log-rank tests were applied to evaluate differences in survival rates. Multivariate Cox proportional hazards models were employed to evaluate associations of molecular subtypes with overall and disease-free survival rates. The following covariates were adjusted in the multivariate models: age at diagnosis, education, income, body mass index (BMI), radiotherapy, chemotherapy, immunotherapy and tamoxifen use, TNM stage, histologic grade, and tumor size. All the tests were performed by using Statistical Analysis Software (SAS, version 9.1; SAS Institute, Inc., Cary, North Carolina). The significance levels were set at P < 0.05 for two-sided analyses.

## Results

Distributions of baseline sociodemographic and clinicopathologic characteristics by 5-year survival rate for the study population of 2,791 subjects are presented in Table 1. The variables significantly related to 5-year survival were: age at diagnosis, education, income, TNM stage, histologic grade and type, ERα, PR, HER2 status, and use of adjuvant therapy (tamoxifen and radiotherapy). The subpopulation of this study was similar to the overall study population for the above characteristics (data not shown).

**Table 1:** Selected demographic and clinical characteristics of breast cancer patients included in the Shanghai Breast Cancer Survival Study.

Variables	Levels	Number of cases	%	5-year survival rate	P
Age at diagnosis	<40	126	4.51	0.9029	<0.01
	40-49	1128	40.42	0.9166	
	50-59	794	28.45	0.8827	
	60-69	469	16.8	0.8586	
	≥70	274	9.82	0.8356	
					
Education	<Middle School	361	12.93	0.7941	<0.01
	Middle School	988	35.40	0.8948	
	> Middle School	1442	51.67	0.9096	
					
Income	<1,000	1723	61.73	0.8696	<0.01
(CNY¥/person/month)	1,000 - 1,999	777	27.84	0.9141	
	≥2,000	291	10.43	0.9415	
					
TNM stage	0	63	2.26	1.0000	<0.01
	I	871	31.21	0.9545	
	IIa	945	33.86	0.9221	
	IIb	549	19.67	0.8174	
	III-IV	272	9.75	0.6724	
					
Histologic grade	I	438	15.69	0.9611	<0.01
	II	1312	47.01	0.9002	
	III	803	28.77	0.8274	
	Unknown	238	8.53	0.9231	
					
Histologic type*	Noninvasive (DCIS, LCIS)	96	3.44	1.0000	<0.01
	ILC NOS	215	7.70	0.8797	
	IDC NOS	1924	68.94	0.8745	
	Special types	527	18.88	0.9264	
	Unknown	29	1.04	0.8966	
					
ERα	Positive (> = 10%)	1740	62.34	0.9206	<0.01
	Negative	1051	37.66	0.8354	
					
PR	Positive (> = 1%)	1632	58.47	0.9183	<0.01
	Negative	1159	41.53	0.8465	
					
HER2	Positive (3+)	849	30.42	0.8621	0.02
	Borderline (2+)	227	8.13	0.8768	
	Negative (0-1+)	1715	61.45	0.9032	
					
Molecular subtype**	Luminal A	1355	48.55	0.9286	<0.01
	Luminal B	467	16.73	0.8862	
	HER2	382	13.69	0.8322	
	Triple negative	360	12.90	0.8069	
	HER2 borderline	227	8.13	0.8768	
					
Radiotherapy	Yes	874	31.31	0.8409	<0.01
	No	1917	68.69	0.9098	
					
Chemotherapy	Yes	2558	91.65	0.8890	0.99
	No	233	8.35	0.8818	
					
Immunotherapy	Yes	477	17.09	0.9118	0.18
	No	2310	82.77	0.8844	
	Unknown	4	0.14	0.7500	
					
Tamoxifen ***	Yes	1484	53.17	0.9123	<0.01
	No	1306	46.79	0.8612	

*DCIS: ductal carcinoma in situ; LCIS: lobular carcinoma in situ; IDC NOS: invasive ductal carcinoma not otherwise specified; ILC NOS: invasive lobular carcinoma not otherwise specified; Special types: including mucinous, papillary, medullary, tubular, cribriform, metaplastic, mixed, and other rare invasive cancers.

** Luminal A: ERα+ and/or PR+, HER2-; luminal B: ERα+ and/or PR+, HER2+; HER2: HER2+, ERα-, and PR-; triple negative: ERα-, PR-, HER2-: HER2 borderline: weak staining of HER2 (2+).

***One case with unknown tamoxifen use was excluded.

Prevalences of the luminal A (ERα + and/or PR+, HER2-), luminal B (ERα + and/or PR+, HER2+), HER2 (HER2+, ERα-, and PR-), and triple-negative (ERα-, PR-, HER2-) subtypes were 48.6%, 16.7%, 13.7%, and 12.9%, respectively. The 8.1% of cases that showed weak positive staining of HER2 (scored as 2+) by IHC were classified as the borderline or equivocal group in this study. During an average of 53.4 months of follow-up after cancer diagnosis, 290 total deaths and 341 recurrences/breast cancer-specific deaths were documented. Differences among molecular subtypes with regard to clinicopathologic characteristics were observed and are presented in Table 2. Significant differences were observed in age at cancer diagnosis (P = 0.03) with luminal A breast cancer being more common among older women (≥70) and triple-negative cancer more common among younger women (<40). Women with the luminal A subtype was more likely to have low TNM stage (P < 0.01), smaller tumor size (P < 0.01), and low histologic grade (P < 0.01) compared with women with the HER2 and triple-negative subtypes. Women with triple-negative breast cancer had a higher frequency of family history of breast cancer than women with other subtypes (P = 0.048).

**Table 2:** Comparisons of clinical and tumor characteristics by molecular subtypes of breast cancer, the Shanghai Breast Cancer Survival Study.

Covariables	Levels	Luminal A	Luminal B	HER2	Triple-negative	HER2 borderline	P value
		N = 1355	N = 467	N = 382	N = 360	N = 227	
		No. (%)	No. (%)	No. (%)	No. (%)	No. (%)	
Mean age at diagnosis*		54.1(10.2)	52.3 (9.3)	53.4 (9.7)	53.5 (10.7)	53.1 (9.9)	0.03
Age at diagnosis							0.02
	<30	3 (0.22)	2 (0.43)	1 (0.26)	4 (1.11)	1 (0.44)	
	30-39	47 (3.47)	21 (4.50)	16 (4.19)	22 (6.11)	9 (3.96)	
	40-49	556 (41.03)	201 (43.04)	138 (36.13)	134 (37.22)	99 (43.61)	
	50-59	364 (26.86)	148 (31.69)	125 (32.72)	97 (26.94)	60 (26.43)	
	60-69	230 (16.97)	63 (13.49)	70 (18.32)	67 (18.61)	39 (17.18)	
	≥70	155 (11.44)	32 (6.85)	32 (8.38)	36 (10.00)	19 (8.37)	
Pre-menopausal		661 (48.78)	239 (51.18)	167 (43.72)	174 (48.33)	114 (50.22)	0.27
Family history of breast cancer		61 (4.50)	21 (4.50)	17 (4.45)	28 (7.78)	17 (7.49)	0.048
TNM stage	0	32 (2.36)	11 (2.36)	8 (2.09)	7 (1.94)	5 (2.20)	<0.01
	I	473 (34.91)	124 (26.55)	91 (23.82)	98 (27.22)	85 (37.44)	
	IIa	452 (33.36)	165 (35.33)	135 (35.34)	122 (33.89)	71 (31.28)	
	IIb	240 (17.71)	98 (20.99)	88 (23.04)	76 (21.11)	47 (20.70)	
	III-IV	109 (8.04)	55 (11.78)	51 (13.35)	42 (11.67)	15 (6.61)	
Tumor size, cm *		2.80 (1.91)	3.31 (3.03)	3.34 (2.26)	3.14 (1.57)	2.82 (1.73)	<0.01
Histologic grade	I	306 (22.58)	31 (6.64)	17 (4.45)	48 (13.33)	36 (15.86)	<0.01
	II	736 (54.32)	217 (46.47)	140 (36.65)	106 (29.44)	113 (49.78)	
	III	219 (16.16)	159 (34.05)	190 (49.74)	175 (48.61)	60 (26.43)	
Histologic type	Noninvasive**	41 (3.03)	20 (4.28)	18 (4.71)	10 (2.78)	7 (3.08)	<0.01
	ILC NOS	142 (10.48)	23 (4.93)	8 (2.09)	18 (5.00)	24 (10.57)	
	IDC NOS	834 (61.55)	361 (77.30)	314 (82.20)	253 (70.28)	162 (71.37)	
	Special types	318 (23.47)	60 (12.85)	39 (10.21)	76 (21.11)	34 (14.98)	

* Number presented as mean (standard deviation)

**Including DCIS and LCIS.

We classified histological types of breast cancer into four categories: non-invasive, invasive lobular, invasive ductal, and invasive special types (data not shown in tables). For non-invasive breast cancer (ductal carcinoma in situ [DCIS] and lobular carcinoma in situ [LCIS]), the most common molecular subtype was luminal A (42.7%), followed by luminal B (20.8%), and the HER2 subtype (18.8%); the triple-negative subtype was least common (10.4%). Among invasive cancers, luminal A accounted for 66% of ILCs, luminal B for 10.7%, the HER2 subtype for 3.7%, and triple-negative for 8.4%. For IDCs, luminal A accounted for 43.3%, luminal B for 18.8%, the HER2 subtype for 16.3%, and triple-negative for 13.1%. Luminal A was the most common molecular subtype among the special histological types of breast cancer (mucinous, 81.2%; papillary, 65.5%; mixed, 58.9%), except for medullary breast cancer where triple-negative was most common (37.9%) (luminal A, 30%; HER2 subtype, 19%).

Associations of molecular subtypes with 5-year overall and disease-free survival rates are presented in Table 3. Women with the luminal A, luminal B, HER2, and triple-negative subtypes had 5-year overall/disease-free survival rates of 92.9/88.6, 88.6/85.1, 83.2/79.1, and 80.7/76.0, respectively. Multivariate Cox regression analyses showed that the HER2 and triple-negative subtypes were associated with an increased risk of overall mortality (hazard ratio (HR) = 1.47, 95% CI, 1.03 to 2.10; HR = 1.87, 95% CI, 1.31 to 2.66, respectively) and breast cancer recurrence/disease-related mortality (HR = 1.32, 95% CI, 0.95 to 1.83; HR = 1.52, 95% CI, 1.09 to 2.11, respectively) after adjustment for age, education, income, BMI, radiotherapy, chemotherapy, immunotherapy, tamoxifen use, TNM stage, histologic grade, and tumor size. A total of 477 cases in our study received some forms of immunotherapy, including IL-2, lymphokine-activated killer cell, and interferon. Immunotherapy was associated with improved overall survival (HR = 0.27, 95% CI, 0.10 to 0.69) and disease-free survival (HR = 0.41, 95% CI, 0.20-0.82) for luminal A breast cancer, but reduced disease-free survival (HR = 2.21, 95% CI, 1.09-4.48) for the HER2 subtype of breast cancer. Use of immunotherapy was not significantly associated with survival for women with luminal B and triple-negative breast cancers.

**Table 3:** Molecular subtypes in association with breast cancer survival, the Shanghai Breast Cancer Survival Study.

	Overall survival					Disease-free survival				
		
	Cases	Events	5-yr survival rate	HR1* (95% CI)	HR2** (95% CI)	Cases	Events	5-yr survival rate	HR1* (95% CI)	HR2** (95% CI)
Luminal A	1258	97	0.9286	1.00	1.00	1234	121	0.8863	1.00	1.00
Luminal B	419	48	0.8862	1.57 (1.10 - 2.22)	1.17 (0.82 - 1.68)	410	57	0.8506	1.42 (1.03 - 1.95)	1.04 (0.75 - 1.43)
HER2	322	60	0.8322	2.12 (1.50 - 3.00)	1.47 (1.03 - 2.10)	312	70	0.7907	1.94 (1.41 - 2.67)	1.32 (0.95 - 1.83)
Triple-negative	298	62	0.8069	2.50 (1.78 - 3.51)	1.87 (1.31 - 2.66)	292	68	0.7600	2.12 (1.55 - 2.91)	1.52 (1.09 - 2.11)
HER2 borderline	204	23	0.8768	1.46 (0.93 - 2.32)	1.42 (0.90 - 2.26)	202	25	0.8788	1.19 (0.77 - 1.84)	1.15 (0.75 - 1.79)

*HR1: Adjusted for age, education, income, BMI, radiotherapy, chemotherapy, immunotherapy, and tamoxifen use.

**HR2: Further adjusted for TNM, histologic grade, and tumor size

## Discussion

### Distribution of molecular subtypes of breast cancer in Chinese women

Our study showed that the prevalence of the triple-negative subtype of breast cancer among Chinese women (12.9%) is similar to that in European populations (10-16%), but lower than in African-American population (20-21%). The HER2 subtype accounted for 13.7% of Chinese breast cancer cases, which is higher than the reported positivity (4-8%) in either European or African-American populations [4,6-8,22,23]. In our study, approximately 8% of breast cancer patients had weak positive or borderline staining (2+) for HER2, which was interpreted as an equivocal category that would be recommended for verification with fluorescent in situ hybridization (FISH) for therapeutic indication of trastuzumab (Herceptin) treatment [15]. The FISH-derived amplification rate for the HER2 equivocal group (i.e., IHC 2+) has been observed to be approximately 25% in Western women [24,25]. If a similar rate is true for Chinese women, most HER2 equivocal cases would fall into either luminal A or triple-negative subtypes. Therefore, the frequencies of luminal A (48.6%) and triple-negative subtypes in our study could be underestimated. We compared ER and PR status for the HER2 borderline group with the HER2+ and HER2- groups, and found that ER+ and PR+ rates (67.4% and 57.7%, respectively) for the HER2 borderline group were more similar to that of the HER2- group (69.7% and 67.1%, respectively) than to the HER+ group (46.1% and 41.3, respectively), suggesting that the vast majority of cases in our HER2 borderline group should likely be classified in the HER2- group. Regardless of the true HER2+ rate for the borderline group, the prevalence of the HER2 subtype in this Chinese population is higher than in Western populations.

The prevalence of breast cancer subtypes appears to differ among different races or ethnicities. It has been well documented that the triple-negative subtype is most common among young African-American patients, while luminal A is most common among postmenopausal white women [4,6-9,22,23]. The increased risk for the triple-negative subtype in African-American women may due to parity and younger age at first full-term pregnancy, multiple live births without breastfeeding, use of medications to suppress lactation [7], and intrinsic genetic variables, such as higher p53 expression [6] and particularly high prevalence of founder mutations in BRCA1 or BRCA2 gene in young (<35 years) African-American women [26]. In our study population, women with triple-negative breast cancer more frequently reported a family history of breast cancer than did women with other subtypes. This suggests that genetic factors may play a more important role in this molecular subtype of breast cancer. Since BRCA mutations in Chinese women are uncommon (1.1% each for BRCA1 and BRCA2) [27]; other genetic contributors to the triple-negative subtype in Chinese women need to be investigated.

We found that HER2+ breast cancers account for 30% of all breast cancer cases in our study population, similar to a previous report from Shanghai (31%) and higher than the reports from Tianjin (26%) [13,14], Taiwan (21%) [28], and the US (26%) [9]. Consistent with our findings, one large, registry-based population study [8] showed that HER2+ tumors are more common among Asian/Pacific Islanders (28%) than among non-Hispanic Whites (21%) or non-Hispanic Blacks (24%), but similar to Hispanics (26%) (Table 4). Another large population study further revealed that among Asian-Americans, Korean and Philipino women had the highest prevalence of HER2+ tumors (36% and 31%, respectively), followed by Vietnamese (29%) and Chinese (26%) women, while Japanese and South Asian women showed a prevalence of HER2+ tumors similar to non-Hispanic Whites and non-Hispanic Blacks (19-23%) [9]. It was not clear why the prevalence of the HER2 subtype or of HER2+ tumors is higher among Chinese or Asian women compared with women of European ancestry or African-American women. Although it has been suggested that environmental factors might play an important role in the etiology of HER2+ breast cancers, variations in criteria used to determine HER2 status may also contribute the differences.

**Table 4:** Distribution of breast cancer subtypes in different ethnicities and in different geographical areas of China, %

References	Ethnicity	Luminal A	Luminal B	HER2	All HER2+*	Triple-negative	Unclassified	**Total No**.
Carey, et al [4]	AA	47.4	12.8	8.2	20.9	26.5	5.1	196
	Caucasian	54.0	17.3	5.7	23.0	16.0	7.0	300
								
Yang, et al [23]	Polish	68.0	14.0	5.0	19.0	13.0		1985
								
Varcone, et al [22]	Italy	68.7	6.0	7.6	13.6	11.8	6.0	804
								
Parise, et al [8]	Non-Hispanic White	67.1	15.1	6.2	21.3	11.6		39051
	Non-Hispanic Black	48.9	14.2	9.8	24.0	27.0		2936
	Hispanic	56.2	16.5	9.7	26.2	17.6		7673
	Asian/Pacific Islander	59.3	18.5	9.9	28.4	12.3		5215
	Other	65.1	14.3	5.4	19.7	15.2		315
								
Telli, et al [9]	Non-Hispanic White	69.6			18.7	11.7		60498
	Non-Hispanic Black	51.1			22.7	26.2		5292
	Hispanic	58.1			24.9	17.0		14106
	Japanese	69.9			19.5	10.7		1136
	Chinese	63.5			25.6	10.8		2305
	Filipino	59.2			30.7	10.1		2802
	Korean	49.4			36.0	14.6		628
	Vietnamese	56.6			29.3	14.1		663
	South Asian	59.2			23.1	17.7		606
	Other Asian**	58.2			28.9	12.9		973
								
Yin, Liu, Lin, et al [10-12]	Chinese (Shanghai)	50.4			31.1	18.5		4787
								
Xing, Zhao, et al [13,14]	Chinese (Tianjin)	53.7	14.0	11.8	25.8	20.5		3237
								
Lin, et al [28]	Chinese (Taiwan)	61.8	8.8	11.8	20.5	12.8	4.9	1028
								
Su, et al (this study)	Chinese (Shanghai)	48.6	16.7	13.7	30.4	12.9	8.1	2791

*All HER2+ tumors include luminal B and HER2 subtypes

**Includes women of Asian Indian, Pakistani, Sri Lankan, and Bangladeshi ethnicity

### Prognostic significance of breast cancer subtypes among Chinese women

Chinese women with the triple-negative subtype were younger in age at diagnosis compared with women who had other subtypes of breast cancer, which is similar to findings reported in Western populations [7,29]. The triple-negative subtype was associated with larger tumor size, higher histologic grade, later TNM stage, and higher prevalence in IDC than in ILC. These clinicopathologic characteristics have been consistently observed in both Western [4,8] and Chinese populations [10-13], suggesting that the triple-negative subtype is an aggressive subtype of breast cancer across all ethnicities. Multivariate analysis confirmed that the triple-negative subtype is an independent prognostic factor for the progression and survival of breast cancer. Most triple-negative cancers defined by IHC present a basal-like subtype profile defined by cDNA microarray, but they do not completely correlate in about 25% of cases [30]. Other molecular subsets may be included in triple-negative cancers. Further epidemiological and biomarker studies for this important subtype in Chinese women is necessary.

The HER2 subtype was closely correlated with larger tumor size and higher histologic grade, consistent with previous reports in other Chinese studies [10-13]. We found that the HER2 subtype was associated with earlier age at diagnosis, more advanced TNM stage, and reduced 5-year overall and disease-free survival rates. Anti-HER2 therapy is currently available. Our study suggests that about 30% of Chinese women with the HER2 subtype (14%) or with the luminal B subtype (17%) may benefit from trastuzumab (Herceptin) and other targeted therapies, if HER2 status were evaluated following the standardized HER2 evaluation guidelines [15] and this information were incorporated into therapeutic decisions.

The luminal B subtype in our study was correlated with younger age at diagnosis, more advanced TNM stage, larger tumor size, higher histologic grade, and was less common in the ILC and special histologic types compared with the luminal A subtype. However, after adjusting for TNM stage, histologic grade, and tumor size, we observed no statistically significant differences for overall or disease-free survival between the two luminal subtypes. Currently, the definition of the luminal B subtype remains debatable. The luminal B subtype originally classified using cDNA microarray gene profiling was unstable and sometimes clustered with the ER- classes (HER2 and basal subtypes) [31,32]. Approximately 30-50% of luminal B class samples defined by gene profiling were HER2+. Therefore, the IHC definition of luminal B (ERα+ and/or PR+, HER2+) is not equivalent to the luminal B tumors classified with microarray gene profiling [4]. Since the gene profile-classified luminal B subtype is defined as tumors with lower expression levels of ERα/PR and related genes, higher proliferative rates, and higher histologic grade [32], some authors have suggested that ERα expression in tumor cells should be semi-quantified using the Allred, Q-score, or H-score to distinguish luminal B from luminal A [33]. More recently, a study [34] suggested that the Ki67 index for cellular proliferation should be combined with ERα, PR, and HER2 to classify luminal tumors into three subtypes: luminal A (ERα+ and/or PR+, HER2-, Ki67 low), luminal B (ERα+ and/or PR+, HER2-, Ki67 high), and luminal-HER2 (ERα+ and/or PR+, HER2+). In that study, the luminal B and luminal-HER2 subtypes had a statistically significant association with poor breast cancer recurrence-free and disease-specific survival in all adjuvant systemic treatment categories. Additional research is warranted to determine the clinical utility of new methods to distinguish luminal breast cancers.

The immune system is thought to play an important role in the metastatic cascade among cancer patients. Thus, various immune strategies have been tested as therapy for breast cancer, including vaccine therapy, administration of exogenous cytokines, monoclonal antibodies, and gene therapy [35]. In our study, we collected general information on immunotherapy by asking participants whether they had received immunotherapies such as IL-2, lymphokine-activated killer (LAK) cell, and interferons. We found that use of immunotherapy was associated with improved overall and disease-free survival among women with the luminal A subtype but with reduced disease-free survival among women with the HER2 subtype, suggesting that choosing the proper immunotherapeutic method should be based on the molecular characteristics of the tumor. This also indicates that analysis of molecular subtypes of breast cancer has significance for personalized immunotherapy to improve the survival of breast cancer patients.

In our study, we found that the molecular subtype of breast cancer is not always consistent with histological type in terms of predicting breast cancer outcomes. For example, in our study, medullary breast cancers accounted for about 19% of the HER2 subtype and 38% of triple-negative cases. Medullary breast cancer is generally considered to be a favorable histological type of breast cancer with a good prognosis. The unfavorable molecular subtypes among medullary breast cancer might not mean an unfavorable outcome. These results suggest that breast cancer is more heterogonous than the four molecular subtypes as defined by ER, PR, and HER2 status. Further investigation into molecular heterogeneity is warranted.

This study is the largest population-based study on molecular subtypes of breast cancer and survival among Chinese women. This study has several notable strengths. The population-based study design and high overall response rate (80%) minimized potential selection bias. Standardized staining and scoring of HER2 status, and centralized pathological confirmation of diagnosis minimized misclassification. There are also some limitations to this study. For example, ERα and PR status for the majority of participants (91% and 92%) was obtained from medical charts. Approximately 8% of cases with borderline positivity for HER2 as determined by IHC were not evaluated with FISH. For cases with missing ERα (234 cases) or PR status (222 cases), ER/PR status was measured at the Vanderbilt centralized laboratory using a cut-off for ER positivity of >10%, which is the cut-off used by the large hospitals in Shanghai [10] and had been validated for the prediction of response to hormonal therapy [20,21]. Due to a slightly decreased PR sensitivity of HER2/PR double staining, a lower cut-off positivity value (>1%) was used for PR positivity. To evaluate the potential influence of the variation in the criteria used to define ER and PR status, we performed additional analysis by excluding cases whose ER and PR status were measured at the centralized laboratory. We did not observe appreciable changes in the study results. In 2010, the American Society of Clinical Oncology/College of American Pathologists recommended that ERα and PR should be considered positive, if there are at least 1% positive tumor nuclei in the tissue samples with proper controls [36]. If the recommended 1% cut-off value for ERα and PR positivity were used in this study, the number of HER2 and triple-negative subtypes would decrease and the number of luminal subtypes would increase. However, the overall prevalence of HER2+ tumors, which includes the luminal B and HER2 subtypes, would not be affected. Future studies on breast cancer subtypes using recommended guidelines [15,36] for hormone receptors and HER2 status are warranted. In addition, the follow-up period of this cohort is relatively short. Our ongoing follow-up with the cohort would overcome this limitation and allow an examination of the long-term effects of different molecular subtypes on the survival of breast cancer patients.

## Conclusions

This large population-based study of Chinese breast cancer survivors confirmed that the triple-negative and HER2+ subtypes were associated with poorer outcomes compared with the luminal A subtype among Chinese women. The HER2+ subtype was more prevalent in this Chinese population compared with Western populations, suggesting the importance of standardized HER2 detection and anti-HER2 therapy to potentially benefit a high proportion of breast cancer patients in China.

## List of abbreviations used

BMI: body mass index; CI: conference interval; DCIS: ductal carcinoma in situ; ERα: estrogen receptor-alpha; ERβ: estrogen receptor-beta; HER2: human epidermal growth factor receptor 2; HR: hazard ratio; IDC: invasive ductal carcinoma; IHC: Immunohistochemistry; ILC: invasive lobular carcinoma; LCIS: lobular carcinoma in situ; PR: progesterone receptor; SBCSS: Shanghai Breast Cancer Survival Study; TMA: tissue microarray.

## Competing interests

The authors declare that they have no competing interests.

## Authors' contributions

XOS, WL, and WZ obtained the funding and designed the study; YS, GL, QC, YZ, and KG acquired the data; YS drafted the manuscript and XOS provided critical review; ZC performed statistical analysis; XOS and WL provided supervision. ALL AUTHORS have reviewed and approved the final version of the paper.

## Pre-publication history

The pre-publication history for this paper can be accessed here:

http://www.biomedcentral.com/1471-2407/11/292/prepub

## Supplementary Material

Additional file 1 **Immunohistochemical double staining method for HER2/PR**. The modified staining protocol for HER2/PR based on the DAKO EnVision™ G|2 Doublestain kit. Click here for file

Additional file 2 **Double immunofluorescence staining method for ERα/ERβ**. The modified double fluorescence staining protocol for ERα/ERβ based on Vector Labs protocol. Click here for file

## References

[B1] Di CS Baselga J Management of breast cancer with targeted agents: importance of heterogenicity Nat Rev Clin Oncol 2010 7 139 147 10.1038/nrclinonc.2009.234 20125090

[B2] van 't Veer LJ Dai H Van de Vijver MJ He YD Hart AA Mao M Peterse HL van der Kooy K Marton MJ Witteveen AT Schreiber GJ Kerkhoven RM Gene expression profiling predicts clinical outcome of breast cancer Nature 2002 415 530 536 10.1038/415530a 11823860

[B3] Sotiriou C Neo SY McShane LM Korn EL Long PM Jazaeri A Martiat P Fox SB Harris AL Liu ET Breast cancer classification and prognosis based on gene expression profiles from a population-based study Proc Natl Acad Sci USA 2003 100 10393 10398 10.1073/pnas.1732912100 12917485 PMC193572

[B4] Carey LA Perou CM Livasy CA Dressler LG Cowan D Conway K Karaca G Troester MA Tse CK Edmiston S Deming SL Geradts J Race, breast cancer subtypes, and survival in the Carolina Breast Cancer Study JAMA 2006 295 2492 2502 10.1001/jama.295.21.2492 16757721

[B5] Nguyen PL Taghian AG Katz MS Niemierko A Abi Raad RF Boon WL Bellon JR Wong JS Smith BL Harris JR Breast cancer subtype approximated by estrogen receptor, progesterone receptor, and HER-2 is associated with local and distant recurrence after breast-conserving therapy J Clin Oncol 2008 26 2373 2378 10.1200/JCO.2007.14.4287 18413639

[B6] Morris GJ Naidu S Topham AK Guiles F Xu Y McCue P Schwartz GF Park PK Rosenberg AL Brill K Mitchell EP Differences in breast carcinoma characteristics in newly diagnosed African-American and Caucasian patients: a single-institution compilation compared with the National Cancer Institute's Surveillance, Epidemiology, and End Results database Cancer 2007 110 876 884 10.1002/cncr.22836 17620276

[B7] Millikan RC Newman B Tse CK Moorman PG Conway K Dressler LG Smith LV Labbok MH Geradts J Bensen JT Jackson S Nyante S Epidemiology of basal-like breast cancer Breast Cancer Res Treat 2008 109 123 139 10.1007/s10549-007-9632-6 17578664 PMC2443103

[B8] Parise CA Bauer KR Brown MM Caggiano V Breast cancer subtypes as defined by the estrogen receptor (ER), progesterone receptor (PR), and the human epidermal growth factor receptor 2 (HER2) among women with invasive breast cancer in California, 1999-2004 Breast J 2009 15 593 602 10.1111/j.1524-4741.2009.00822.x 19764994

[B9] Telli ML Kurian AW Chang ET Keegan TH Ford JM Gomez SL Asian race and breast cancer subtypes: A study from the California Cancer Registry Breast Cancer Res Treat 2011 127 471 478 10.1007/s10549-010-1173-8 20957431 PMC4349378

[B10] Liu ZB Liu GY Yang WT Di GH Lu JS Shen KW Shen ZZ Shao ZM Wu J Triple-negative breast cancer types exhibit a distinct poor clinical characteristic in lymph node-negative Chinese patients Oncol Rep 2008 20 987 994 18813844

[B11] Yin WJ Lu JS Di GH Lin YP Zhou LH Liu GY Wu J Shen KW Han QX Shen ZZ Shao ZM Clinicopathological features of the triple-negative tumors in Chinese breast cancer patients Breast Cancer Res Treat 2009 115 325 333 10.1007/s10549-008-0096-0 18563552

[B12] Lin Y Yin W Yan T Zhou L Di G Wu J Shen Z Shao Z Lu J Site-specific relapse pattern of the triple negative tumors in Chinese breast cancer patients BMC Cancer 2009 9 342 10.1186/1471-2407-9-342 19778431 PMC2760577

[B13] Zhao J Liu H Wang M Gu L Guo X Gu F Fu L Characteristics and prognosis for molecular breast cancer subtypes in Chinese women J Surg Oncol 2009 100 89 94 10.1002/jso.21307 19544363

[B14] Xing P Li J Jin F A case-control study of reproductive factors associated with subtypes of breast cancer in Northeast China Med Oncol 2010 27 926 931 10.1007/s12032-009-9308-7 19771534

[B15] Wolff AC Hammond ME Schwartz JN Hagerty KL Allred DC Cote RJ Dowsett M Fitzgibbons PL Hanna WM Langer A McShane LM Paik S American Society of Clinical Oncology/College of American Pathologists guideline recommendations for human epidermal growth factor receptor 2 testing in breast cancer Arch Pathol Lab Med 2007 131 18 43 19548375 10.5858/2007-131-18-ASOCCO

[B16] Shu XO Zheng Y Cai H Gu K Chen Z Zheng W Lu W Soy food intake and breast cancer survival JAMA 2009 302 2437 2443 10.1001/jama.2009.1783 19996398 PMC2874068

[B17] Su Y Shrubsole MJ Ness RM Cai Q Kataoka N Washington K Zheng W Immunohistochemical expressions of Ki-67, cyclin D1, beta-catenin, cyclooxygenase-2, and epidermal growth factor receptor in human colorectal adenoma: a validation study of tissue microarrays Cancer Epidemiol Biomarkers Prev 2006 15 1719 1726 10.1158/1055-9965.EPI-05-0946 16985035

[B18] Tavassoli FA Devilee P World Health Organization Classification of Tumours. Pathology and Genetics of Tumours of the Breast and Female Genital Organs 2003 London, IARC Press

[B19] Rakha EA El-Sayed ME Lee AH Elston CW Grainge MJ Hodi Z Blamey RW Ellis IO Prognostic significance of Nottingham histologic grade in invasive breast carcinoma J Clin Oncol 2008 26 3153 3158 10.1200/JCO.2007.15.5986 18490649

[B20] Diaz LK Sahin A Sneige N Interobserver agreement for estrogen receptor immunohistochemical analysis in breast cancer: a comparison of manual and computer-assisted scoring methods Ann Diagn Pathol 2004 8 23 27 10.1016/j.anndiagpath.2003.11.004 15129906

[B21] Fisher ER Anderson S Dean S Dabbs D Fisher B Siderits R Pritchard J Pereira T Geyer C Wolmark N Solving the dilemma of the immunohistochemical and other methods used for scoring estrogen receptor and progesterone receptor in patients with invasive breast carcinoma Cancer 2005 103 164 173 10.1002/cncr.20761 15565575

[B22] Zarcone M Amodio R Campisi I Cusimano R Dolcemascolo C Miceli V Traina A Macaluso M Application of a new classification to a breast tumor series from a population-based cancer registry: demographic, clinical, and prognostic features of incident cases, Palermo Province, 2002-2004 Ann N Y Acad Sci 2009 1155 222 226 10.1111/j.1749-6632.2008.03693.x 19250207

[B23] Yang XR Sherman ME Rimm DL Lissowska J Brinton LA Peplonska B Hewitt SM Anderson WF Szeszenia-Dabrowska N Bardin-Mikolajczak A Zatonski W Cartun R Differences in risk factors for breast cancer molecular subtypes in a population-based study Cancer Epidemiol Biomarkers Prev 2007 16 439 443 10.1158/1055-9965.EPI-06-0806 17372238

[B24] Lebeau A Deimling D Kaltz C Sendelhofert A Iff A Luthardt B Untch M Lohrs U Her-2/neu analysis in archival tissue samples of human breast cancer: comparison of immunohistochemistry and fluorescence in situ hybridization J Clin Oncol 2001 19 354 363 11208826 10.1200/JCO.2001.19.2.354

[B25] Perez EA Roche PC Jenkins RB Reynolds CA Halling KC Ingle JN Wold LE HER2 testing in patients with breast cancer: poor correlation between weak positivity by immunohistochemistry and gene amplification by fluorescence in situ hybridization Mayo Clin Proc 2002 77 148 154 10.4065/77.2.148 11838648

[B26] John EM Miron A Gong G Phipps AI Felberg A Li FP West DW Whittemore AS Prevalence of Pathogenic BRCA1 Mutation Carriers in 5 US Racial/Ethnic Groups JAMA 2007 298 2869 2876 10.1001/jama.298.24.2869 18159056

[B27] Suter NM Ray RM Hu YW Lin MG Porter P Gao DL Zaucha RE Iwasaki LM Sabacan LP Langlois MC Thomas DB Ostrander EA BRCA1 and BRCA2 mutations in women from Shanghai China Cancer Epidemiol Biomarkers Prev 2004 13 181 189 10.1158/1055-9965.EPI-03-0196 14973102

[B28] Lin CH Liau JY Lu YS Huang CS Lee WC Kuo KT Shen YC Kuo SH Lan C Liu JM Kuo WH Chang KJ Cheng AL Molecular subtypes of breast cancer emerging in young women in Taiwan: evidence for more than just westernization as a reason for the disease in Asia Cancer Epidemiol Biomarkers Prev 2009 18 1807 1814 10.1158/1055-9965.EPI-09-0096 19505913

[B29] Kwan ML Kushi LH Weltzien E Maring B Kutner SE Fulton RS Lee MM Ambrosone CB Caan BJ Epidemiology of breast cancer subtypes in two prospective cohort studies of breast cancer survivors Breast Cancer Res 2009 11 R31 10.1186/bcr2261 19463150 PMC2716499

[B30] Ismail-Khan R Bui MM A review of triple-negative breast cancer Cancer Control 2010 17 173 176 20664514 10.1177/107327481001700305

[B31] Loi S Sotiriou C Buyse M Rutgers E Van't Veer L Piccart M Cardoso F Molecular forecasting of breast cancer: time to move forward with clinical testing J Clin Oncol 2006 24 721 722 10.1200/JCO.2005.04.6524 16446348

[B32] Sorlie T Tibshirani R Parker J Hastie T Marron JS Nobel A Deng S Johnsen H Pesich R Geisler S Demeter J Perou CM Repeated observation of breast tumor subtypes in independent gene expression data sets Proc Natl Acad Sci USA 2003 100 8418 8423 10.1073/pnas.0932692100 12829800 PMC166244

[B33] Bhargava R Dabbs DJ Luminal B breast tumors are not HER2 positive Breast Cancer Res 2008 10 404 10.1186/bcr2134 18831725 PMC2614503

[B34] Cheang MC Chia SK Voduc D Gao D Leung S Snider J Watson M Davies S Bernard PS Parker JS Perou CM Ellis MJ Ki67 index, HER2 status, and prognosis of patients with luminal B breast cancer J Natl Cancer Inst 2009 101 736 750 10.1093/jnci/djp082 19436038 PMC2684553

[B35] Florescu A Amir E Bouganim N Clemons M Immune therapy for breast cancer in 2010-hype or hope? Curr Oncol 2011 18 e9 e18 21331271 10.3747/co.v18i1.623 PMC3031364

[B36] Hammond ME Hayes DF Dowsett M Allred DC Hagerty KL Badve S Fitzgibbons PL Francis G Goldstein NS Hayes M Hicks DG Lester S Love R Mangu PB McShane L Miller K Osborne CK Paik S Perlmutter J Rhodes A Sasano H Schwartz JN Sweep FC Taube S Torlakovic EE Valenstein P Viale G Visscher D Wheeler T Williams RB Wittliff JL Wolff AC American Society of Clinical Oncology/College of American Pathologists Guideline Recommendations for Immunohistochemical Testing of Estrogen and Progesterone Receptors in Breast Cancer J Clin Oncol 2010 28 2784 2795 10.1200/JCO.2009.25.6529 20404251 PMC2881855

