# Supplementary material for: Distinct distribution and prognostic significance of molecular subtypes of breast cancer in Chinese women: a population-based cohort study
Source: BMC Cancer. 2011 Jul 12;11:292. doi: 10.1186/1471-2407-11-292 (PMC3157458; doi:10.1186/1471-2407-11-292)
Supplement: Additional file 1 — Immunohistochemical double staining method for HER2/PR. The modified staining protocol for HER2/PR based on the DAKO EnVision™ G|2 Doublestain kit. [file 1471-2407-11-292-S1.DOC]

**Additional files**

**Additional file 1. Immunohistochemical double staining method for HER2/PR.**

The sections were deparaffinized, and antigen retrieval was performed by heating the slides with a pressure cooker (2100 Retriever, PickCell Lab.) in EDTA buffer (pH 8.0, Zymed). Slides then were immunostained following the procedure of the EnVision™ G|2 Doublestain kit (DAKO, Cat# K5361). Rabbit polyclonal antibody recognizing HER2 cytoplasmic domain (DAKO, Cat# A0485, 1:100) was stained with DAB+ chromogen (brown in nuclei), and rabbit monoclonal antibody recognizing the PR N-terminus (Epitomics, Clone Y85, Cat# 1483-1, 1:20) was stained with Permanent Red chromogen for 20 min (red in cytoplasm). Both colors allowed for visualization against a hematoxylin counterstain (Figure 1). The known PR/HER2 positive breast carcinoma tissue was used as positive control, and replacing primary antibodies with PBS was used as negative control. The intensity of HER2 and PR was validated by comparing standard HER2 single staining and PR single staining, which was conducted with the same rabbit monoclonal anti PR antibody (Epitomics, 1:100) following the protocol of DAKO Envision^TM^ kit (DAKO, Cat# K4011).
