# Supplementary material for: Distinct distribution and prognostic significance of molecular subtypes of breast cancer in Chinese women: a population-based cohort study
Source: BMC Cancer. 2011 Jul 12;11:292. doi: 10.1186/1471-2407-11-292 (PMC3157458; doi:10.1186/1471-2407-11-292)
Supplement: Additional file 2 — Double immunofluorescence staining method for ERα/ERβ. The modified double fluorescence staining protocol for ERα/ERβ based on Vector Labs protocol. [file 1471-2407-11-292-S2.DOC]

**Additional files**

**Additional file 2. Double immunofluorescence staining method for ERα/ERβ.**

We used a modified version of the Vector Labs protocol: <http://www.vectorlabs.com/infopage.asp?dpID=53&locID=684325> for double immunoflourescence staining of ERα/PRβ. The sections were deparaffinized and antigen retrieval was performed by heating the slides with a pressure cooker in 10 mM citrate buffer. For staining of the first antigen, avidin/biotin blocking and protein blocking steps were performed with an Avidin/Biotin Blocking Kit (Vector, Cat# SP-2001) and 5% normal goat serum (Vector, Cat# S-1000), respectively. We incubated the slides with the first primary antibody (mouse monoclonal anti-ERβ recognizing the amino acids 1-153 of human, Abcam, Clone 14C8, Cat# ab288, 1:200) for 1 hr at RT, biotinylated goat anti-mouse Ab (Vector, Cat# BA-9200, 5ug/ml) for 30 min, and fluorescein avidin DCS (Vector, Cat# A-2011, 15 ug/ml) for 10 min. For staining of the second antigen, we repeated the avidin/biotin blocking and protein blocking steps as before, incubating slides with the second primary antibody (rabbit monoclonal anti-ERα recognizing C-terminal portion, LabVision, Clone SP1, Cat# RM-9101, 1:100) for 1 hr at RT, biotinylated goat anti-rabbit Ab (Vector, Cat# BA-1000, 5ug/ml) for 30 min, and Texas red avidin DCS (Vector, Cat# A-2016, 15 ug/ml) for 10 min. We washed the slides in buffer between each step. The slides were coverslipped with the anti-fade and anti-photobleaching VECTASHIELD HardSet Mounting Medium with DAPI (Vector, Cat# H-1500). The intensity of ERα and ERβ staining was validated by comparing with standard single staining, conducted with the same rabbit anti-ERα antibody (LabVision, 1:200) following the protocol of the DAKO Envision^TM^ rabbit kit (DAKO, Cat# K4011), and with the same mouse monoclonal anti-ERβ antibody (Abcam, 1:200) following the protocol of the DAKO Envision^TM^ mouse kit (DAKO, Cat# K4006) (Figure 2). The lab-constructed TMA-positive control slides were stained in parallel with each batch. Nuclear ERβ was green, nuclear ER-α was red, nuclei with expression of both ERs was yellow, and nuclei negative for both ERs were counterstained with DAPI in blue (Figure 3). Replacement of the primary antibodies with PBS was used as a negative control.
